# Supplementary material for: The causal relationship between the human gut microbiota and pyogenic arthritis: a Mendelian randomization study
Source: Front Cell Infect Microbiol. 2024 Nov 26;14:1452480. doi: 10.3389/fcimb.2024.1452480 (PMC11629706; doi:10.3389/fcimb.2024.1452480)
Supplement: Supplementary file 1 [file DataSheet1.zip › mendelian test/ebi-a-GCST90027603.csv_leave_one_out.pdf]

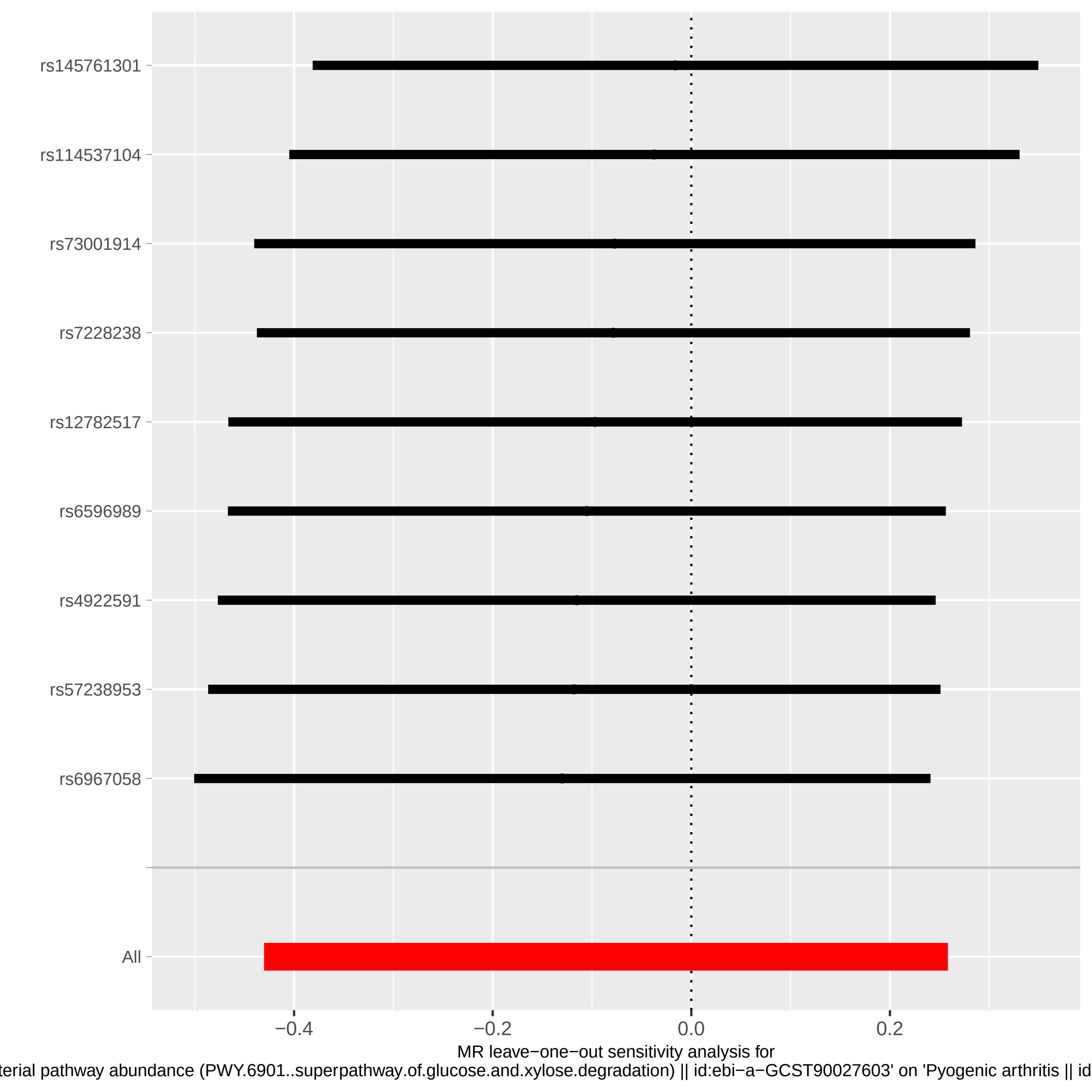

rs145761301
rs114537104
rs73001914
rs7228238
rs12782517
rs6596989
rs4922591
rs57238953
rs6967058
All
−0.4
−0.2
0.0
0.2
MR leave−one−out sensitivity analysis for
terial pathway abundance (PWY.6901..superpathway.of.glucose.and.xylose.degradation) || id:ebi−a−GCST90027603' on 'Pyogenic arthritis || id
